# Evaluating the effectiveness of oral phlebotonics for the conservative management of hemorrhoidal disease: insights from the VIVI2022/01/VIVALDI study

**DOI:** 10.3389/fsurg.2026.1771489

**Published:** 2026-05-12

**Authors:** Iacopo Giani, Fulvio Leopardi, Tommaso Bonetti, Renato Pietroletti, Gaetano Gallo, Leonardo Tersigni, Erica Tanasi, Roberto Piazza, Mario Mangrella, Claudio Elbetti

**Affiliations:** 1 SOSD Proctologia, USL Toscana Centro, Firenze, Italy; 2 Unità Semplice Dipartimentale di Chirurgia Proctologica e del Perineo, Azienda Ospedaliera Universitaria Integrata di Verona, Verona, Italy; 3 Department of Applied Clinical and Biotechnological Sciences, University of L'Aquila Surgical Coloproctology Hospital Val Vibrata, Sant'Omero, Italy; 4 Colorectal Surgery Unit, IRCCS San Raffaele Scientific Institute, Vita-Salute University, Milan, Italy; 5 Italfarmaco SpA, Medical Affairs Department, Milano, Italy

**Keywords:** enhanced recovery after surgery (ERAS), flavonoids, hemorrhoidal disease symptom score (HDSS), hemorrhoidal disease, phlebotonics, *Ruscus aculeatus*, short health scale adapted for HD, single pile hemorrhoid classification (SPHC), *Vitis vinifera*

## Abstract

**Background:**

The aim of this study is to describe the baseline characteristics of an Italian, real-world cohort of patients with symptomatic hemorrhoidal disease (HD) and to evaluate the effectiveness and safety of phlebotonic drugs, in addition to standard of care.

**Methods:**

This is a prospective, multicenter, observational cohort study (VIVI2022/01/VIVALDI, ClinicalTrials.gov number: NCT07376928) on adults with symptomatic Goligher grade I-II-III HD. The primary endpoint was the improvement in the hemorrhoidal disease symptom score (HDSS). Secondary endpoints were based on the resolution of bleeding (Giamundo score), improvement in quality of life (Short Health Scale adapted for HD), Goligher grading, Single Pile Hemorrhoid Classification (SPHC), adverse events, satisfaction with treatment, and change in treatment strategy during the 30-day observational period. Patients were evaluated by visits and questionnaires at baseline and after 30 days; at day 30, the evaluation also included the assessment of surgical indication. At day 15, patients were also interviewed through a phone call in order to verify adherence and unexpected effects.

**Results:**

A total of 115 patients (70% male), with a mean (SD) age of 51.4 years and HD diagnosed since an average of 4.6 (8.3) years, were enrolled. The mean HDSS decreased from 7.8 (3.6) at baseline to 3.7 (2.6) at day 30 (*p* < 0.001). Bleeding was reported by 94.8% of patients at baseline vs. 58.3% at day 30 (*p* < 0.0001). At day 30, 39.1% of patients displayed a Goligher downstaging. The mean SHS-HD score decreased from 11.29 (3.8) at baseline to 7.56 (2.8) at day 30 (*p* < 0.0001). The average number of piles per patient was 2.72 (1.6) at baseline and 2.51 (1.3) at day 30 [mean change: −0.32 (1.42); *p* = 0.0026]. Only one patient (<1%) reported one non-serious adverse event (moderate abdominal distension), which was deemed not related to the studied drug and did not lead to treatment discontinuation during the 30-day follow-up. Treatment was deemed satisfactory by 73% of patients, with none discontinuing within day 30.

**Conclusion:**

A 30-day course of conservative therapy with oral phlebotonics was found to be safe and effective in improving a variety of clinical measures in adults with symptomatic Goligher grade I-II-III HD.

## Introduction

Hemorrhoidal disease (HD) is non-seasonal and prevalent on a global scale (1) affecting 4.4%–86% of patients (2), with bleeding being the most significant and frequently reported symptom.

Several changes in the physiological regulation of vein homeostasis are involved in the pathophysiology of HD, including mechanical injury to the anal cushions, pathological venous dilatation, venous stasis (with or without thrombosis), inflammation, and degenerative collagen fiber deposition (1, 3). HD affects health-related quality of life (QoL), and this seems to be linked to the severity of symptoms (4) but not to the Goligher Classification (5, 6).

Hemorrhoidectomy is considered the gold standard for the definitive treatment of high-grade symptomatic HD (7). However, due to the wide range of symptoms, an ever-increased attention has been paid to the surgical strategy. In this context, over the years, different surgical techniques have been integrated in the same patient (8, 9) as part of a holistic approach. At the same time, the role of medical therapy has been reevaluated, with phlebotonics and conservative measures (lifestyle modifications, high-fiber diets, stool softeners, laxatives, and sitz baths) being found to be useful in all HD Goligher grades. This conservative approach is currently preferred for the first-line management of grade I to III HD (2, 10), both as a bridge to surgery and as a consolidation of the hemorrhoidal tissue before and after any surgical approach.

Phlebotonic agents, such as the flavonoid diosmin, constitute a diverse class of drugs, whose mechanism of action is not yet completely elucidated. It has been hypothesized that these agents are capable of enhancing the integrity of blood vessel walls, improve venous tone and lymphatic drainage, regulate capillary permeability, and exert anti-inflammatory effects (4, 10–13). Flavonoids have been identified as potentially effective for reducing preoperative signs and symptoms, improving Goligher grade and decreasing indication to surgery (14–16), and minimizing postoperative pain and complications, regardless of the severity of HD (17).

The extracts of *Vitis vinifera* (18), *Ruscus aculeatus* (19), and *Melilotus officinalis* (20) have been documented to individually support the regular functionality of microcirculation, venous circulation, and the hemorrhoidal plexus, as well as hesperidin, quercetin, and bromelain (18, 21, 22).

The aim of this VIVI2022/01/VIVALDI study is to describe the baseline characteristics of an Italian, real-world cohort of patients with symptomatic Goligher grade I, II, or III HD and to evaluate the effectiveness and safety of phlebotonic drugs, as a conservative treatment in patients with symptomatic I to III grade HD.

## Methods

The VIVI2022/01/VIVALDI study (ClinicalTrials.gov number, NCT07376928) is an Italian, multicenter, prospective, observational cohort study designed to collect data on adults with symptomatic Goligher grade I, II, or III HD (23, 24). Patients received conservative therapy of HD with oral phlebotonics, prescribed at the clinician's discretion within a real-world clinical practice setting, along with standard of care (SOC, i.e., high-fiber diet, laxatives, removal of risk factors, and improvement of bowel habits.). All treatments administered during the study adhered to standard practices and guidelines (10).

The study was approved by the following ethics committees: Comitato Etico Regionale per la Sperimentazione Clinica della Regione Toscana Sezione: AREA VASTA CENTRO (prot 23683_oss) on 21 February 2023; Comitato etico CESC delle Province di Verona e Rovigo (Prot. n. 33325 del 5 June 2023) on 31 May 2023; Regione Calabria—Comitato Etico Territoriale—(Registro Protocollo n. 60 del 9 novembre 2023) on 9 November 2023; Comitato Etico Territoriale Regione Abruzzo—C.Et.R.A (Verbale n°13) on 25 January 2024. All enrolled patients provided written informed consent before any study procedures were undertaken, and the study was conducted in accordance with the principles of Good Clinical Practice and the Declaration of Helsinki.

Patients were included in the study if they were aged ≥18 years and able to give signed informed consent, had symptomatic I–III HD grade, and were about to start treatment with phlebotonics (either in case they never used phlebotonics in the past or if they had stopped any phlebotonic since at least 1 month). Exclusion criteria were the presence of history of colorectal or anal cancer and/or radiation therapy, obstructive defecation syndrome, irritable bowel syndrome, inflammatory bowel disease, coagulation disorders, other relevant proctologic diseases such as anal abscess or fistula, anal fissure, or acute hemorrhoidal thrombosis, ongoing treatment with anticoagulant or antiaggregant drugs, and pregnancy or breastfeeding.

The primary study endpoint was the improvement of the hemorrhoidal disease symptom score (HDSS) (25).

Secondary endpoints were bleeding reduction measured through the Giamundo score (26), improvement in quality of life evaluated through the Short Health Scale adapted for HD (SHS-HD) (27), downstaging according to the Goligher Classification (23), improvement of the clinical picture assessed by the Single Pile Hemorrhoid Classification (SPHC) (28), patient satisfaction with treatment through a 5-point Likert-type scale (Very Unsatisfied, Unsatisfied, Neutral, Satisfied, Very Satisfied) (29), and change in treatment strategy during and at the end of the 30-day observational period.

The safety secondary endpoint was the proportion of patients with serious and non-serious adverse events (AEs/ADRs/SAEs and SADRs) during the administration of oral conservative therapies. Patients were examined, and clinical signs and symptoms were reported and assessed through the scores described above, at baseline and at day 30. The baseline and day 30 proctological visit included the following: clinical history, anal inspection, dynamic clinical examination during strain and rest, digital rectal examination, and anoscopy. At day 30, the evaluation also included the assessment of surgical indication. Patients were also interviewed at day 15 through a phone call, in order to verify adherence and untoward effects.

A nested analysis in patients treated with diosmin, *Vitis vinifera* extract, *Ruscus aculeatus*, and *Melilotus officinalis* is presented in addition to that of the overall population.

### Statistical analysis

Quantitative data were reported as mean (standard deviation, SD), while categorical variables were reported as number (*n*) and percentage (%). Changes in continuous variables between baseline and day 30 were analyzed using the paired Wilcoxon signed rank test and McNemar's test was used for examining categorical variables.

A two-sided *p* value < 0.05 was considered statistically significant for all analyses, which were carried out using SAS® Software, v 9.4 (SAS Institute, Cary, NC, USA).

## Results

Between May 2023 and December 2024, 115 patients (70.4% males), with a mean (SD) age of 51.4 (14.5) years (range 20–86 years), were enrolled in the study. The mean age at HD diagnosis was 46.8 (16.1) years and the disease duration was 4.6 (8.3) years. Full details of the patients’ baseline characteristics are illustrated in Table 1.

**Table 1:** Baseline characteristics of participants.

Characteristic	Overall sample (*N* = 115)
Male (*n*, %)	81 (70.4%)
Age, years (mean, SD)	51.43 (14.5)
Caucasian ethnicity (*n*, %)	113 (98.3%)
Age at HD diagnosis, years (mean, SD)	46.82 (16.1)
Time since HD diagnosis, years (mean, SD)	4.61 (8.3)
At least one current treatment for diseases other than HD (*n*, %)	33 (28.7%)
At least one current non-phlebotonic treatment for HD (*n*, %)	13 (11.3%)
Goligher grade HD (*n*, %)	
Grade I = No prolapse	16 (13.9%)
Grade II = Prolapse on defecation with spontaneous reduction	55 (47.8%)
Grade III = Prolapse on defecation requiring manual reduction	44 (38.3%)

HD, hemorrhoidal disease; SD, standard deviation.

Only 13 patients (11.3%) were treated with a drug (the combination of diosmin and hesperidin), while 102 (88.7%) were treated with food supplements (the combination of diosmin, extract of *Vitis vinifera*, *Ruscus aculeatus*, *Melilotus officinalis*, and the combination of diosmin, hesperidin, quercetin, casein hydrolysate, bromelain, and *Ruscus aculeatus*), in addition to SOC.

### Hemorrhoidal disease symptom score

**Figure 1:**
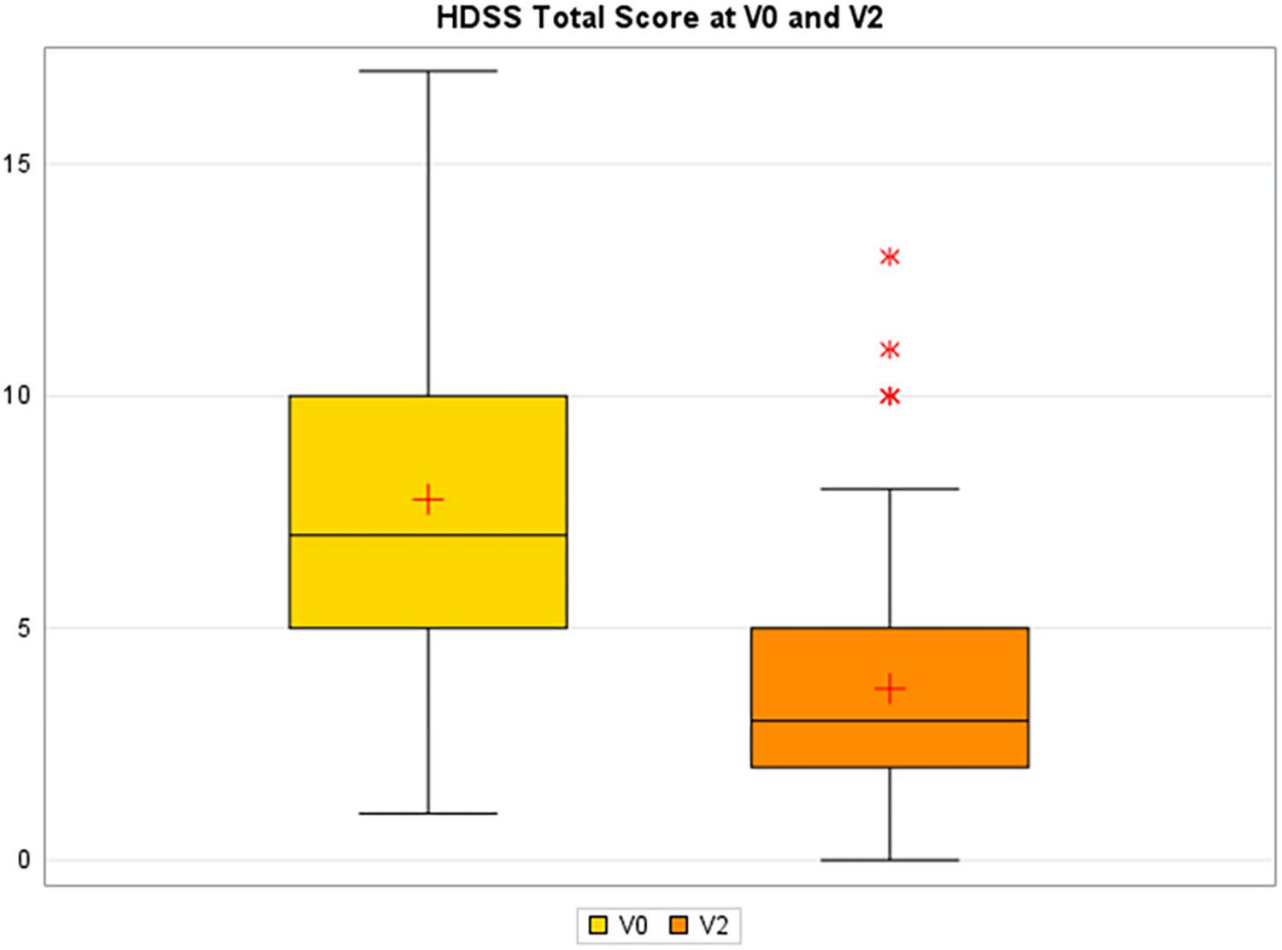
Total hemorrhoidal disease symptom score (HDSS) at baseline and day 30. The central black line within the boxes represents the median, while the box itself covers the interquartile range, indicating the 25th (Q1) to 75th (Q3) percentiles of the data. The whiskers extend to the smallest and largest non-outlier. The red plus signs indicate the mean total HDSS for each visit. V0 = baseline. V2 = Day 30.

The mean total HDSS decreased significantly from 7.8 (3.6) points at baseline to 3.7 (2.6) points at day 30, with a mean change of −4.08 (2.8) points (*p* < 0.0001) (Figure 1). The HDSS at baseline and at day 30 are reported in detail in Table 2: they show a general reduction in symptom severity.

**Table 2:** Hemorrhoidal disease symptom score (HDSS) at the studied time points.

Question		Baseline, % (*n*/*N*)	Day 30, % (*n*/*N*)
Question 1. How often do you feel pain from your hemorrhoids?	Never	55.7 (64/115)	80.9 (93/115)
	Less than once a month	24.3 (28/115)	14.8 (17/115)
	Less than once a week	12.2 (14/115)	3.5 (4/115)
	1–6 days per week	7.8 (9/115)	0.9 (1/115)
Question 2. How often do you feel itching or discomfort in the anus?	Never	26.1 (30/115)	40.9 (47/115)
	Less than once a month	22.6 (26/115)	34.8 (40/115)
	Less than once a week	17.4 (20/115)	16.5 (19/115)
	1–6 days per week	18.3 (21/115)	3.5 (4/115)
	Every day (always)	15.7 (18/115)	4.3 (5/115)
Question 3. How often do you bleed when passing stool?	Never	5.2 (6/115)	46.1 (53/115)
	Less than once a month	19.1 (22/115)	21.7 (25/115)
	Less than once a week	18.3 (21/115)	20.0 (23/115)
	1–6 days per week	36.5 (42/115)	8.7 (10/115)
	Every day (always)	20.9 (24/115)	3.5 (4/115)
Question 4. How often do you soil your underwear (soiling from the anus)?	Never	67.0 (77/115)	77.4 (89/115)
	Less than once a month	7.8 (9/115)	12.2 (14/115)
	Less than once a week	6.1 (7/115)	8.7 (10/115)
	1–6 days per week	12.2 (14/115)	0.9 (1/115)
	Every day (always)	7.0 (8/115)	0.9 (1/115)
Question 5. How often do you feel a swelling or a prolapsing hemorrhoid?	Never	27.0 (31/115)	45.2 (52/115)
	Less than once a month	15.7 (18/115)	25.2 (29/115)
	Less than once a week	13.9 (16/115)	12.2 (14/115)
	1–6 days per week	20.0 (23/115)	7.8 (9/115)
	Every day (always)	23.5 (27/115)	9.6 (11/115)

Remarkably, the percentage of patients who never bleed when passing stool increased from 5.2% at baseline to 46.1% at day 30, the percentage of those never feeling itching or discomfort increased from 26.1% at baseline to 40.9% at day 30, the percentage of patients never feeling pain improved from 55.7% at baseline to 80.9% at day 30, and the percentage of those never feeling a swelling or a prolapsing hemorrhoid improved from 27% at baseline to 45.2% at day 30.

### Bleeding entity measured through the Giamundo score

**Figure 2:**
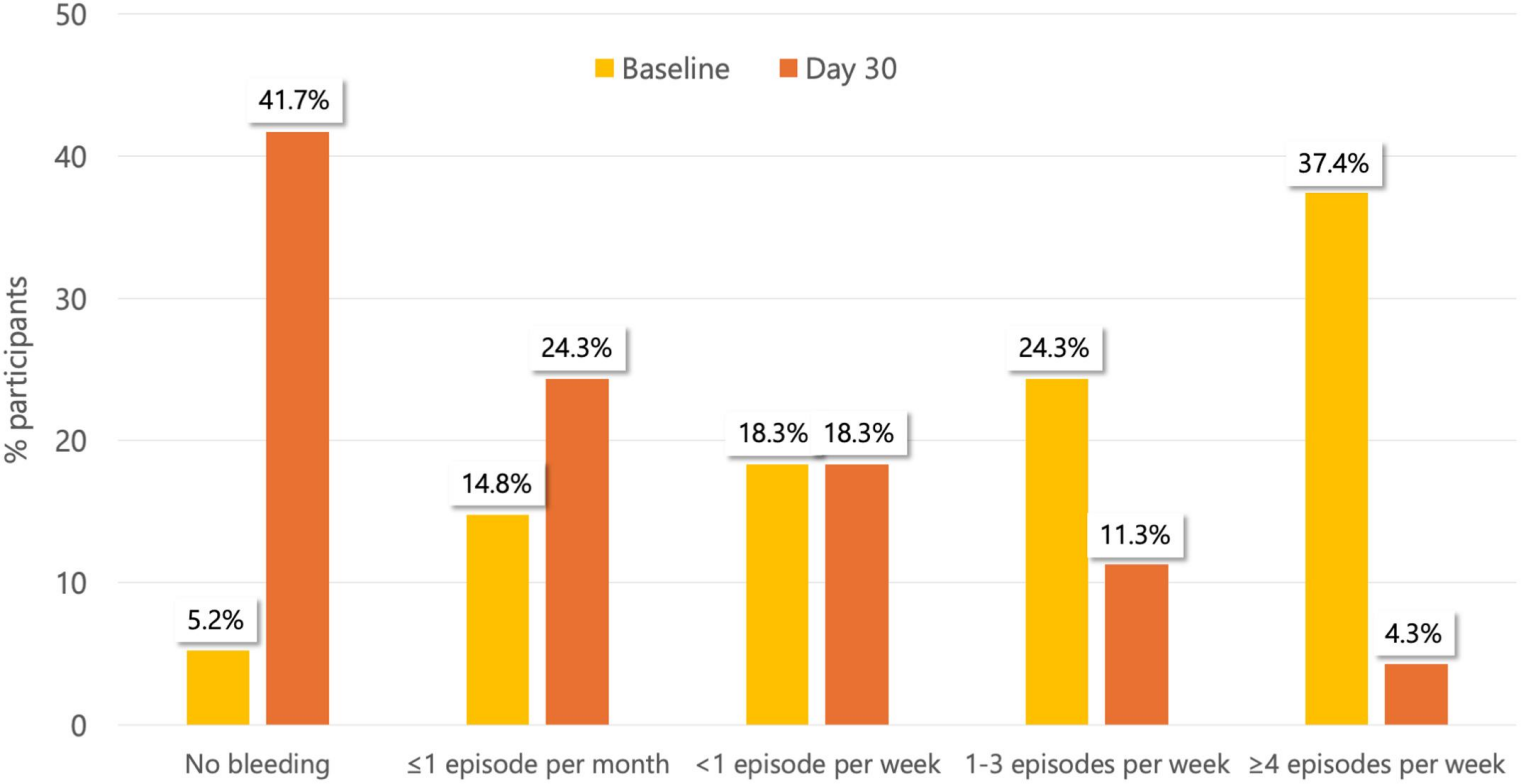
Bleeding as assessed by the Giamundo score at the study time points.

Consistently, there was a significant reduction in bleeding measured through the Giamundo score at day 30 vs. baseline (*p* < 0.0001). In particular, 109/115 (94.8%) patients had bleeding (any grade) at baseline vs. 67/115 (58.3%) at day 30. Remarkably, 41.7% (48/115) of participants had no bleeding at day 30 vs. only 5.2% (6/115) at baseline and only 4.3% (5/115) claimed ≥4 episodes per week at day 30 vs. 37.4% (43/115) at baseline (Figure 2).

**Figure 3:**
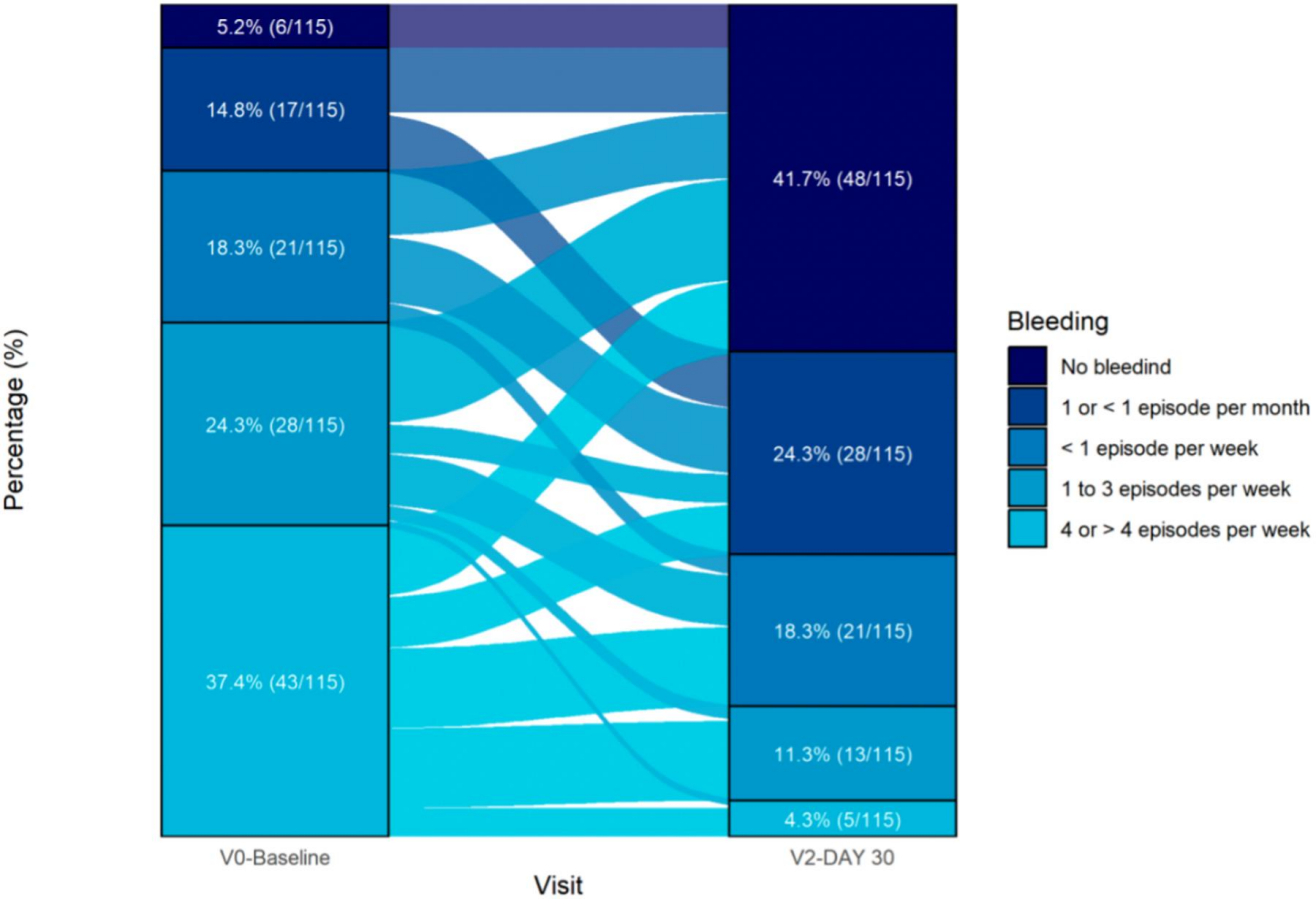
Alluvial plots of bleeding transitions from baseline to day 30.

Figure 3 shows alluvial plots of bleeding transitions from baseline to day 30, according to the Giamundo score. In detail, all patients with no bleeding at baseline had no bleeding also at day 30. Nine out of 17 (52.9%) of those with one episode or less per month at baseline had no bleeding at day 30 and 8/17 (47.1%) had the same frequency of bleeding also at day 30. Among the 21 patients with less than one episode of bleeding per week at baseline, 9 (42.9%) had no bleeding at day 30, 9 (42.9%) claimed one episode or less per month, and 3 (14.3%) claimed the same frequency of bleeding also at day 30. Among the 28 patients with up to three episodes of bleeding per week at baseline, 14 (50%) had no bleeding, four (14.3%) claimed only one or less episode per month, seven (25%) less than one episode per week, one (3.6%) an increase to four or more episodes per week at day 30, and two (7.1%) reported the same frequency of bleeding also at day 30. Finally, among the 43 patients with four or more episode of bleeding per week at baseline, 10 (23.3%) had no bleeding at day 30, 7 (16.3%) reported a reduction to one or less episode per month, 11 (26.6%) claimed less than one episode per week, 11 (26.6%) 1–3 episodes per week, and four (9.3%) still claimed four or more episodes of bleeding per week at day 30.

### Goligher classification

**Figure 4:**
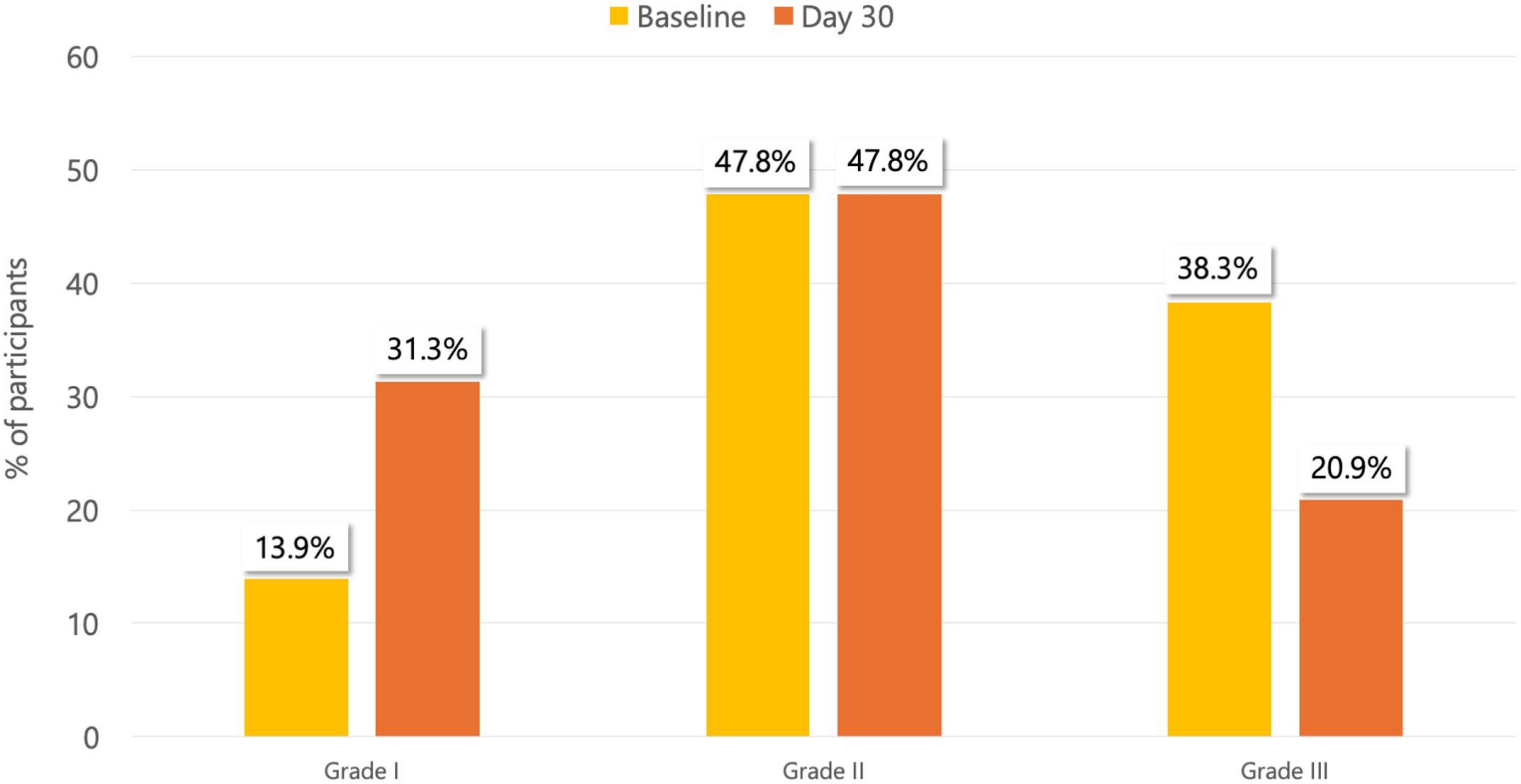
Goligher grading at the study time points. Grade I = No prolapse; Grade II = Prolapse on defecation with spontaneous reduction; Grade III = Prolapse on defecation requiring manual reduction.

The Goligher grading at day 30, compared with baseline, is reported in Figure 4. A downstaging at day 30 from baseline in the Goligher grade was observed in 45/115 (39.1%) patients (*p* < 0.0001): among 44 patients with Goligher grade III HD at baseline, 24 (54.5%) improved to grade II and 2 (4.5%) improved to grade I at day 30; 19/55 (34.5%) of those with Goligher grade II HD at baseline improved to grade I at day 30. In 63/115 (54.8%) patients, there was no change in Goligher grading between baseline and day 30, and in 7/115 (6.1%), a worsening from baseline of Goligher grading at day 30 was observed (Table 3).

**Table 3:** Change in the Goligher grade from baseline to day 30.

Change in the Goligher grade from baseline to day 30	Overall sample (*N* = 115) % (*n*/*N*)
From I to I	13.0 (15/115)
From I to II	0.9 (1/115)
From II to I	16.5 (19/115)
From II to II	26.1 (30/115)
From II to III	5.2 (6/115)
From III to I	1.7 (2/115)
From III to II	20.9 (24/115)
From III to III	15.7 (18/115)

### Short health scale adapted for hemorrhoidal disease

An improvement in the SHS-HD score was also detected: the mean total score decreased significantly from 11.29 (3.8) at baseline to 7.56 (2.8) at day 30 (*p* < 0.0001). Answers to the SHS-HD questionnaire are provided in Table 4.

**Table 4:** Short health scale adapted for hemorrhoidal disease (SHS-HD) score at the study time points.

Question	Baseline (*n* = 115) mean (SD)	Day 30 (*n* = 115) mean (SD)
In your view, how severe are your symptoms caused by hemorrhoids?	3.15 (1.3)	2.08 (0.9)
Do your symptoms interfere with your daily activities?	2.69 (1.6)	1.83 (1.1)
Do your symptoms cause much concern?	3.09 (1.6)	1.69 (1.0)
How is your general feeling of wellbeing?	2.37 (1.4)	1.96 (1.0)
Total score	11.29 (3.8)	7.56 (2.8)

SD, standard deviation.

### Single pile hemorrhoid classification

The information on the SPHC was recorded both at baseline and at day 30 in 109 patients, for a total of 296 pathological piles at baseline and 259 at day 30. The average number of piles per patient was 2.72 (1.6) at baseline and 2.51 (1.3) at day 30 [mean change: −0.32 (1.42); *p* = 0.0026]. The full details of the SPHC are given in Table 5.

**Table 5:** Single pile hemorrhoid classification (SPHC) at the study time points.

Characteristic		Baseline	Day 30	*p*-Value
Goligher grade	I	29.4% (87/296)	46.7% (121/259)	<0.0001^a^
	II	46.6% (138/296)	41.7% (108/259)	
	III	24.0% (71/296)	11.2% (29/259)	
	IV	0.0% (0/296)	0.4% (1/259)	
Fibrous inelastic redundant pile (F)	*N* (yes); median (Q1–Q3)	21; 1.00 (1.00–2.00)	21; 0.00 (0.00–1.00)	0.0510^b^
Subversion of the dentate line or congestion of external pile (E)	*N* (yes); median (Q1–Q3)	25; 1.00 (1.00–2.00)	25; 1.00 (0.00–1.00)	0.0181^b^
Not tolerated skin tags (S)	*N* (yes); median (Q1–Q3)	25, 1.00 (1.00–2.00)	25, 0.00 (0.00–0.00)	<0.0001^b^
Signs of hemorrhoidal inflammation	*N* (yes); median (Q1–Q3)	88; 2.00 (1.00–3.00)	88; 1.00 (0.00–1.50)	<0.0001^b^
Bleeding	*N* (yes); median (Q1–Q3)	73; 1.00 (1.00–2.00)	73; 0.00 (0.00–1.00)	<0.0001^b^

a From the McNemar–Bowker test.

b From the Wilcoxon signed rank test.

### Grade of satisfaction

The grade of satisfaction with the medical therapy received (as assessed by the use of a Likert 5-point scale) was high: 84/115 (73.1%) of patients declared themselves satisfied or very satisfied, only 3/115 (2.6%) were unsatisfied or very unsatisfied, while the remaining 28/115 (24.3%) gave a neutral opinion on treatment.

### Safety and posttherapy strategy

One patient (<1%) reported one non-serious adverse event (moderate abdominal distension), which was deemed not related to the studied drug and did not lead to treatment discontinuation during the 30-day follow-up. None had serious adverse events. At day 30, 38 patients received indication to surgery.

### Outcomes of patients treated with the combination of diosmin, extract of *Vitis vinifera*, *Ruscus aculeatus*, and *Melilotus officinalis*

Among the 82 patients treated with diosmin, *Vitis vinifera* extract, *Ruscus aculeatus*, and *Melilotus officinalis*, characteristics and treatment outcomes closely matched those of the overall study population. Fifty-nine (72%) were male patients; their mean (SD) age was 50.16 (14.39) years, and their disease duration was 4.62 (8.67) years. By the Goligher classification, 12.2% had no prolapse, 50% had prolapse on defecation with spontaneous reduction, and 37.8% had prolapse on defecation requiring manual reduction.

**Figure 5:**
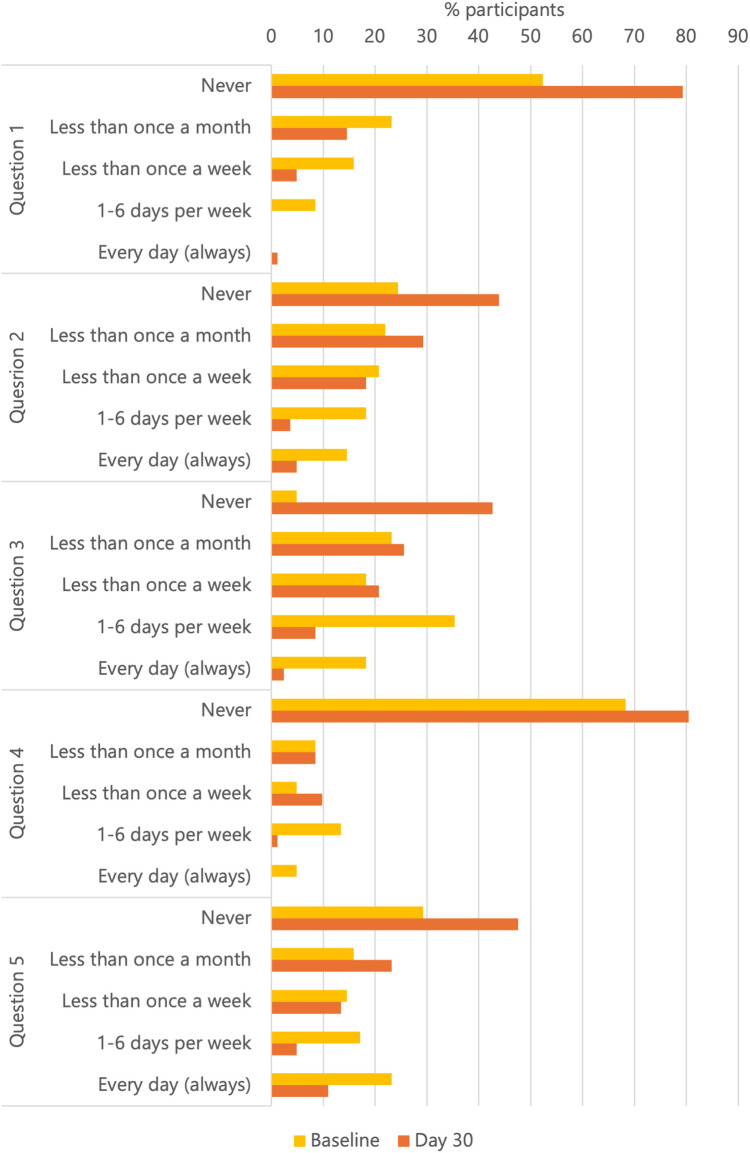
Hemorrhoidal disease symptom score (HDSS) single items at baseline and day 30 in the 82 patients treated with the combination of diosmin, extract of *Vitis vinifera*, *Ruscus aculeatus*, and *Melilotus officinalis*. Question 1—Pain. Question 2—Itching or Discomfort. Question 3—Bleeding. Question 4—Soil. Question 5—Swelling or Prolapsing.

A significant reduction in the HDSS between baseline and day 30 was observed [mean HDSS at baseline: 7.63 [3.82] points; mean HDSS at day 30: 3.68 [2.75] points; mean change: −3.95 [2.97] points; *p* < 0.0001]. The percentage of patients who never bleed when passing stool increased from 4.9% at baseline to 42.7% at day 30, the percentage of those never feeling itching or discomfort increased from 24.4% at baseline to 43.9% at day 30, the percentage of patients never feeling pain improved from 52.4% at baseline to 79.3% at day 30, and the percentage of those never feeling a swelling or a prolapsing hemorrhoid improved from 29.3% at baseline to 47.6% at day 30 (Figure 5). An improvement in the Goligher grade was observed in 31/82 (37.8%) patients (*p* = 0.0004); in particular, 17/31 (54.8%) of patients with grade III at baseline showed a downstaging. There was a parallel significant reduction in bleeding assessed through the Giamundo score at day 30 vs. baseline (*p* < 0.0001): in detail, 78/82 (95.1%) patients reported bleeding at baseline vs. 51/82 (62.3%) at day 30. The average number of pile per patient was 2.74 (1.39) at baseline and 2.56 (1.45) at day 30. An improvement in the SHS-HD score was also detected: the mean total score decreased significantly from 11.24 (4.03) at baseline to 7.6 (2.82) at day 30 (*p* < 0.0001).

Only one (1.2%) patient who received the combination of diosmin, *Vitis vinifera* extract, *Ruscus aculeatus*, and *Melilotus officinalis* declared being unsatisfied, while 76% (62/82) declared being satisfied or very satisfied with the treatment.

## Discussion

This study showed that phlebotonics were safe, well-tolerated, and able to reduce symptoms and health-related QoL in patients with Goligher grade I to III HD. The HDSS was our primary endpoint due to its ability to differentiate between high and low symptom burden (25). The main study finding was clearly the improvement in this score, but an equally significant finding was that the symptom reduction achieved through these pharmacological interventions was consistently validated by other assessment scores, including SHS-HD, Giamundo bleeding score, Goligher grading, and SPHC.

There was a significant reduction in bleeding episodes/severity, with 46.1% of individuals experiencing no bleeding on day 30 compared with 5.2% at baseline. In addition, the downstaging of the Goligher grade was clinically relevant, which was observed in 39.1% of participants. While not directly comparable, this finding aligns with the outcomes of a retrospective study involving 49 individuals with grade II and III HD (22). The evidence of a clinical downstaging of HD represents an important outcome, which has impact both on symptoms and potentially on the surgical pathway, as well as postsurgical healing. However, the Goligher classification has some limitations: it considers only internal piles behavior and defines different groups according to the most prolapsed pile, but it does not describe the severity of symptoms. By contrast, the SPHC considers the number of pathological piles and the characteristics of each internal and external pile (28). With regard to the internal pile, the SPHC considers the Goligher grade and the presence of a fibrous inelastic redundant pile; with regard to the external pile, it takes into account the presence of the subversion of the dentate line or the congestion of the external pile as well as the presence of intolerable skin tags. All these anorectal abnormalities may contribute to symptoms and need to be considered for the management of treatment options. As compared to the Goligher classification, the SPHC might thus represent a more in-depth evaluation of HD and even seems to be a better tool for directing the surgical strategy (28). Therefore, particularly relevant are the improvements in the SPHC that we observed during this study. In fact, in addition to a statistically significant downstaging in the Goligher grade and bleeding, we observed statistically significant reductions in fibrous inelastic redundant piles, in the subversion of the dentate line or congestion of external piles, in not tolerated skin tags, and in signs of hemorrhoidal inflammation (8, 9, 28). Altogether, the results of this study suggest that flavonoids could have a rightful place among the clinical tools of prehabilitation, in the context of the all-encompassing approach known as the “enhanced recovery after surgery (ERAS) pathway” (30, 31).

The results presented here strongly suggest a significant improvement in health-related QoL after just 30 days of treatment with the tested medical approaches. Indeed, an enhancement in the HDSS was directly associated with improved QoL levels in patients with HD (4).

Notably, medical treatments were very well tolerated: only one patient reported one non-serious adverse event (deemed not related to the studied drug) and 73% of patients expressed satisfaction with the therapy received.

Most patients received treatment with food supplements. Therefore, the study results predominantly reflect the efficacy and safety of this class of phlebotonics, and specifically of the combination of diosmin, *Vitis vinifera* extract, *Ruscus aculeatus*, and *Melilotus officinalis*. Consistently, treatment outcomes among patients who received this combination closely matched that of the overall study population. Among patients treated with this combination, there was approximately a 10-fold reduction in the proportion of those with ≥4 bleeding episodes per week and a 7-fold increase in the proportion of those with no bleeding at day 30 compared with baseline. Furthermore, a downstaging of the Goligher grade was observed in the majority of patients with grade III at baseline, and there was a strong reduction (approximately an halving) of the global HDSS.

The phlebotonic properties of flavonoids are well documented, although their specific mechanism of action remains debated (10, 32–35). Despite limitations in methodological quality, heterogeneity, and potential publication bias, a meta-analysis involving 14 trials and 1,514 randomized patients suggested that flavonoids decrease by 58% the risk of non-reducing or persisting symptoms and also apparently reduce the risk of bleeding (RR 0.33), persistent pain (RR 0.35), itching (RR 0.65), and recurrence of symptoms (RR 0.53) (2). In addition, a recent Consensus Statement of the Italian Society of Colorectal Surgery on the management and treatment of HD acknowledged that the conservative treatment of HD includes phlebotonics such as flavonoids (10).

The findings of the present study are consistent with those of the majority of clinical trials, which have demonstrated that flavonoids are effective in HD and yield significantly better outcomes than placebo (11, 12, 16, 36–38). Although not specifically investigated, this makes it unlikely that the results of this study are related to a placebo effect of the treatments that the patients received.

A notable strength of this study is that we sought a comprehensive evaluation of the effectiveness of various phlebotonics, taking into account both patient perception through patient-reported outcome measures (PROMs) and objective clinical data and using multiple rating scales recently introduced in the literature but still rarely used currently in clinical practice (39, 40).

It is important, however, to also acknowledge certain limitations of our study. Primarily, the absence of randomization in treatment allocation and the imbalance between treatment groups hindered our ability to make comparisons across different medical interventions with adequate statistical power. Furthermore, the study design did not include a control group and the treatments were heterogeneous. However, it must be emphasized that this was only an exploratory investigation intended to evaluate the feasibility of planning larger, prospective, comparative trials. In addition, it should be recognized that the results of this study are applicable only to patients with Goligher grades I–III HD. Furthermore, individuals suffering from systemic diseases that may be associated with and influence the progression of HD were excluded from this study. This limitation restricted our sample size and the generalizability of our results. Consequently, the findings cannot provide information on the efficacy and safety of the study treatment in patients with more severe HD or systemic diseases associated with HD. Finally, due to the scheduling of a 30-day follow-up, this study was unable to determine the optimal duration for this treatment.

Notwithstanding the aforementioned limitations, the findings presented herein indicate that a conservative management of Goligher grades I to III HD may ameliorate both symptomatic presentation and anatomical abnormalities associated with hemorrhoids, pending surgical intervention. The treatments evaluated in this study could thus have the potential to act as a bridge to surgery. In this sense, a prospective study specifically designed to assess the efficacy of phlebotonic therapies as a bridge to surgery in patients with advanced HD showed a significant reduction in both symptoms and all inflammatory signs following treatment (41). Therefore, flavonoid-based therapies should be regarded as effective even when surgery remains indicated, as they can optimize the hemorrhoidal condition prior to surgical intervention even in patients with more advanced HD.

In conclusion, a 30-day course of conservative medical therapy of Goligher grade I-II-III HD with oral phlebotonics was found to be safe and associated with improvement in a variety of clinical measures (HDSS, Goligher grade, Giamundo bleeding score, SHS-HD, SPHC). The enhancement in these clinical parameters was corroborated by the health-related QoL score SHS-HD, and patient satisfaction with the evaluated treatments was notably high. The findings of this study also emphasize the need for randomized controlled trials with longer follow-up times in this clinical area. Finally, the outcomes of this study predominantly reflect the efficacy and safety of the combination of diosmin, *Vitis vinifera* extract, *Ruscus aculeatus*, and *Melilotus officinalis*.

## Data Availability

The raw data supporting the conclusions of this article will be made available by the authors to qualified researchers upon reasonable request, contingent on a scientifically sound research proposal.
